# Supplementary material for: Identification of Trichomonas vaginalis 5-Nitroimidazole Resistance Targets
Source: Pathogens. 2023 May 10;12(5):692. doi: 10.3390/pathogens12050692 (PMC10223654; doi:10.3390/pathogens12050692)

Figure S1. Gene ontology enrichment word clouds for 134 upregulated genes. Molecular function; Biological process; Cellular component.

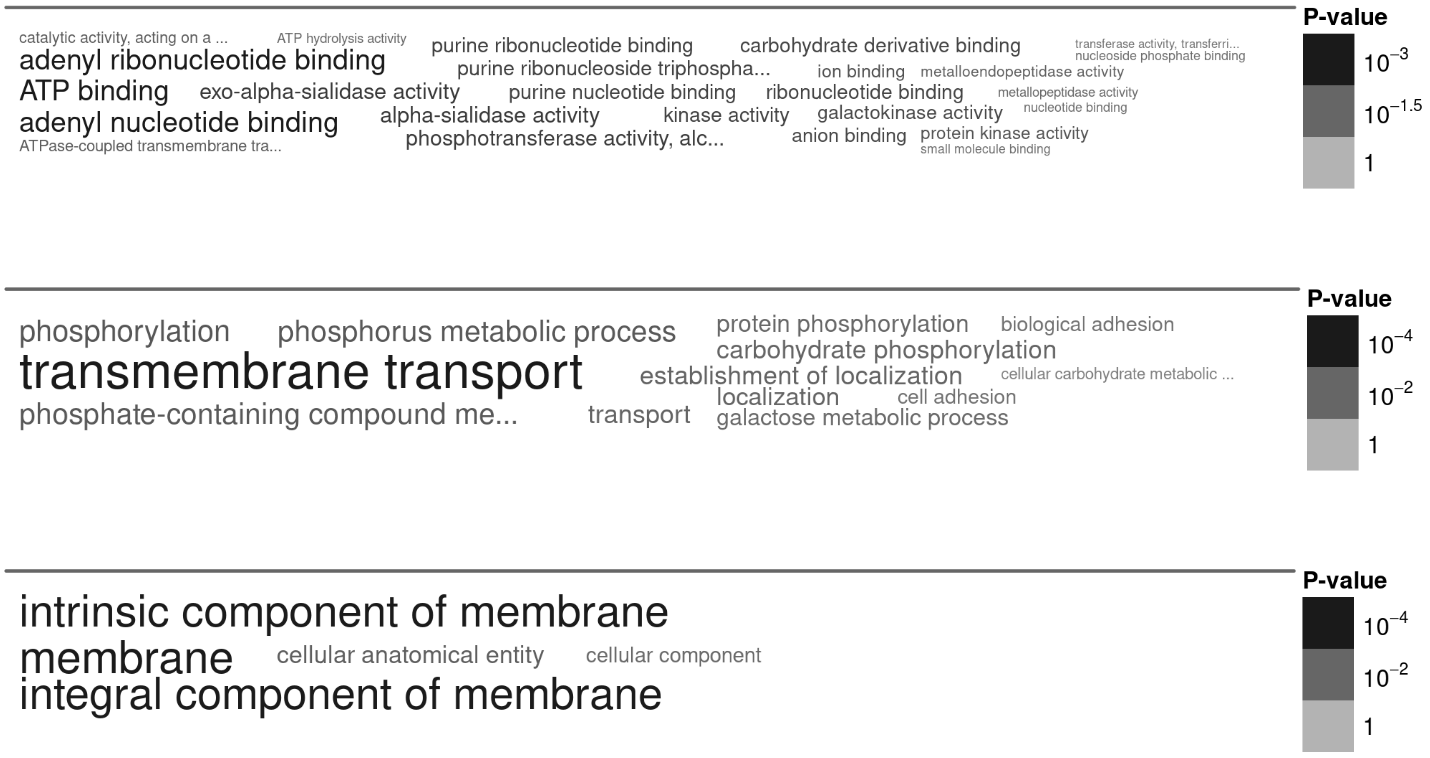

Figure S2. Gene ontology enrichment word clouds for 170 downregulated genes. Molecular function; Biological process; Cellular component.

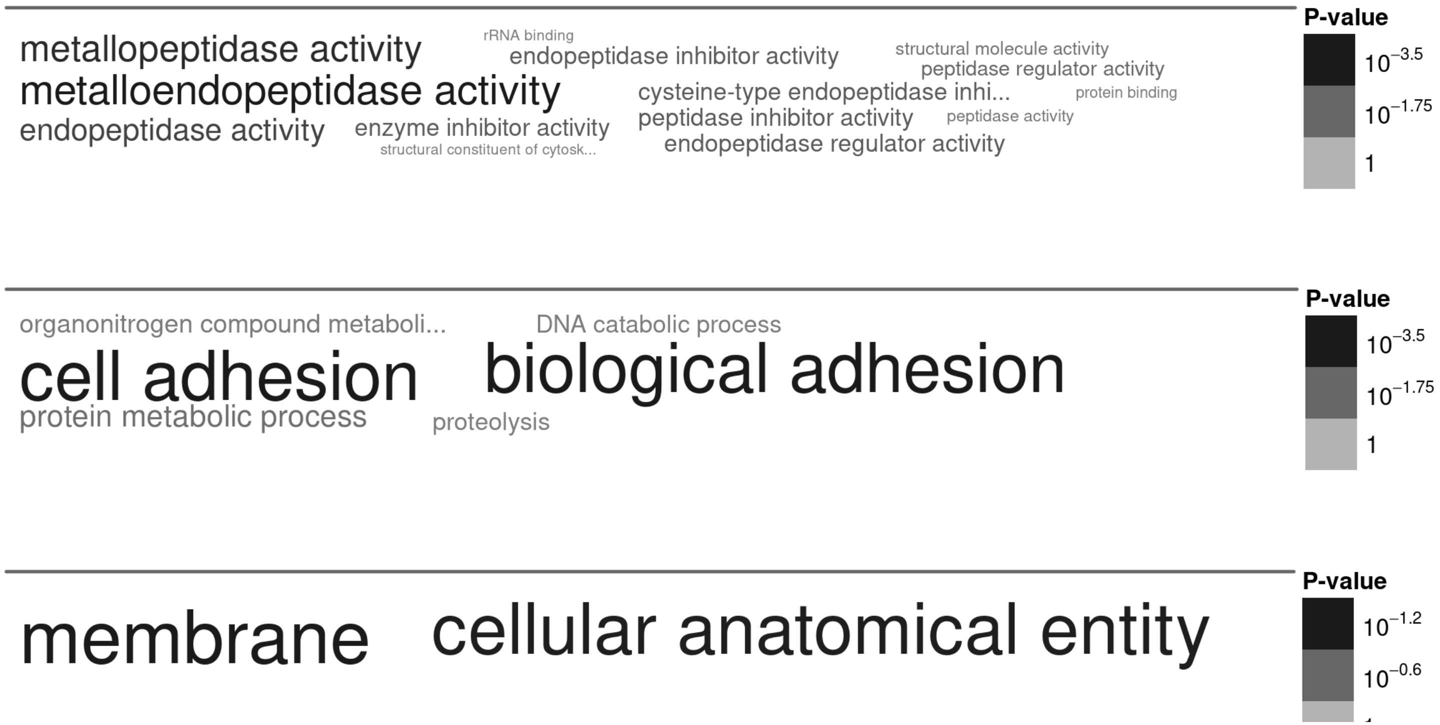

Supplement: Supplementary file 1 [file pathogens-12-00692-s001.zip › FiguresS1-S2_GO.pdf]
