# Supplementary material for: Identification of Trichomonas vaginalis 5-Nitroimidazole Resistance Targets
Source: Pathogens. 2023 May 10;12(5):692. doi: 10.3390/pathogens12050692 (PMC10223654; doi:10.3390/pathogens12050692)
Supplement: Supplementary file 1 [file pathogens-12-00692-s001.zip › Table_S1_RNA.Purity_revised.pdf]

Table S1. Total RNA quality of *T. vaginalis* isolates

Isolate	Purity (A260/A280)	Concentration (ng/µl)
252-1	2.01	1762.2
252-2	1.85	1059.5
252-3	1.92	1101.2
4266-1	1.82	326.3
4266-2	1.42	1919.7
4266-3	2.00	1624.3
4448-1	1.98	1539.8
4448-2	2.04	1521.8
4448-3	1.91	1707.7
1073-1	1.94	1060.8
1073-2	1.85	1800.6
1073-3	1.79	383.2
904-1	1.96	1241.1
904-2	1.98	1852.2
904-3	1.91	837.1
1003-1	2.02	813.8
1003-2	2.03	1784.1
1003-3	2.01	1275.7
1012-1	2.07	1603.8
1012-2	2.01	1514.3
1012-3	1.38	1871.9
1021-1	2.01	1274.5
1021-2	1.53	1963.3
1021-3	0.99	1708.6
